# Impact of on-site cardiac catheterization on resource utilization and fatal and non-fatal outcomes after acute myocardial infarction

**DOI:** 10.1186/1472-6963-6-148

**Published:** 2006-11-10

**Authors:** Abdul R Halabi, Christine A Beck, Mark J Eisenberg, Hugues Richard, Louise Pilote

**Affiliations:** 1 Divisions of Cardiology, Montreal General Hospital, McGill University Health Center, Montreal, Canada; 2 Clinical Epidemiology, Montreal General Hospital, McGill University Health Centre, Montreal, Canada; 3 Divisions of Cardiology and Clinical Epidemiology, Jewish General Hospital, Montreal, Canada

## Abstract

**Background:**

Patterns of care for acute myocardial infarction (AMI) strongly depend on the availability of on-site cardiac catheterization facilities. Although the management found at hospitals without on-site catheterization does not lead to increased mortality, little it known about its impact on resource utilization and non-fatal outcomes.

**Methods:**

We identified all patients (n = 35,289) admitted with a first AMI in the province of Quebec between January 1, 1996 and March 31, 1999 using population-based administrative databases. Medical resource utilization and non-fatal and fatal outcomes were compared among patients admitted to hospitals with and without on-site cardiac catheterization facilities.

**Results:**

Cardiac catheterization and PCI were more frequently performed among patients admitted to hospitals with catheterization facilities. However, non-invasive procedures were not used more frequently at hospitals without catheterization facilities. To the contrary, echocardiography [odds ratio (OR), 2.04; 95% confidence interval (CI), 1.93–2.16] and multi-gated acquisition imaging (OR, 1.24; 95% CI, 1.17–1.32) were used more frequently at hospitals with catheterization, and exercise treadmill testing (OR, 1.02; 95% CI, 0.91–1.15) and Sestamibi/Thallium imaging (OR, 0.93; 95% CI, 0.88–0.98) were used similarly at hospitals with and without catheterization. Use of anti-ischemic medications and frequency of emergency room and physician visits, were similar at both types of institutions. Readmission rates for AMI-related cardiac complications and mortality were also similar [adjusted hazard ratio, recurrent AMI: 1.02, 95% CI, 0.89–1.16; congestive heart failure: 1.02; 95% CI, 0.90–1.15; unstable angina: 0.93; 95% CI, 0.85–1.02; mortality: 0.99; 95% CI, 0.93–1.05)].

**Conclusion:**

Although on-site availability of cardiac catheterization facilities is associated with greater use of invasive cardiac procedures, non-availability of catheterization did not translate into a higher use of non-invasive tests or have an impact on the fatal and non-fatal outcomes available for study in our administrative database.

## Background

Advances in pharmacological therapy and mechanical revascularization have markedly contributed to improved outcomes following acute myocardial infarction (AMI). However, practice variations persist with respect to the use of these treatment strategies. One factor known to contribute to this variation is the regionalization of facilities for invasive cardiac procedures. Strong, positive associations between the use of invasive cardiac procedures and the presence of on-site cardiac catheterization facilities have been established [1-9].

Several clinical trials and observational studies have suggested that a more invasive approach to management of AMI – as is found at hospitals with on-site cardiac catheterization facilities – is not associated with improved survival for all patient subgroups [2-4,6,7,10-17]. Thus, it is possible that the benefit of a universally applied invasive approach may not be commensurate with its substantial use of resources. However, it is also possible that there are aspects of a more selectively invasive approach – as is found at hospitals without on-site cardiac catheterization – that drive resource utilization, such as the use of non-invasive procedures after AMI.

We sought to determine how availability of on-site cardiac catheterization facilities at the hospital of admission for AMI might influence resource utilization, as well as fatal and non-fatal outcomes after AMI. We hypothesized that non-availability of cardiac catheterization facilities might translate into a higher use of non-invasive cardiac procedures, given the need to perform cardiac risk stratification. We also hypothesized that a lower use of invasive cardiac procedures might impact on the use of anti-ischemic medications, emergency room and outpatient physician visits, readmissions for AMI-related complications and mortality.

## Methods

### Study Population

A cohort of AMI patients was constructed from the universal hospital discharge summary database for the province of Quebec, Canada. We identified all patients admitted with a discharge diagnosis of AMI [International Classification of Diseases (ICD), 9^th ^revision code 410] between January 1^st ^1996 and March 31^st ^1999 (n = 40,857). The positive predictive value for coding an AMI for elderly patients in the database has been evaluated to be 96% (95% CI, 94–98) [18]. In order to increase comparability and include patients based on only first episodes of AMI, we excluded patients who had a previous diagnosis of AMI between 1988 and 1995 (9.3%). Additional patients were excluded if they were admitted to non-cardiac surgical units, or if the total length of stay (including transfers) for their admission was less than three days, because AMI is unlikely to be the primary diagnosis for such patients. Following these exclusions, our study population consisted of 35,289 patients (total exclusions were 13.6% of the original cohort). This method to identify AMI patients has been used extensively in Quebec [19], Ontario [20], and the United States [21].

Using encrypted Quebec Medicare numbers, we further linked the discharge summary database with the physician and drug claims database for the province of Quebec. These databases contain information on all diagnostic and therapeutic procedures in Quebec. They also contain information on resource use, such as outpatient physician visits and outpatient prescriptions filled. Due to the characteristics of the Quebec health care and health insurance systems, information on prescriptions was limited to patients 65 years of age and older.

Mortality data were obtained by merging data from several different sources: the hospital discharge summary database, institutions that manage pensions, government human resources and car insurance, and provincial death certificates. The algorithm we created using these different sources of information allowed us to determine the vital status for close to 100% of the study patients. All patients were followed from the date of their initial admission until December 31, 1999.

### Hospital Groups

Study patients were grouped based on whether their admitting hospital had on-site cardiac catheterization facilities. A total of 16 hospitals had on-site cardiac catheterization facilities over the study period, and there were no changes in availability of catheterization at each of these hospitals over the study period. However, the number of hospitals without cardiac catheterization facilities changed over time, as there were some hospital closures between 1996 and 1999. Over these years, 100 to 116 hospitals were hospitals without cardiac catheterization facilities. Patients were classified according to whether or not they were initially admitted for AMI at hospitals with or without cardiac catheterization facilities. If a patient was initially admitted to a hospital without catheterization, and then transferred to a hospital with catheterization, the patient was "assigned" to the initial hospital without catheterization only.

### Medical Care Utilization

For each patient, we obtained data on all in- and outpatient invasive and non-invasive cardiac procedures performed during the follow-up period. The invasive procedures studied were cardiac catheterization, percutaneous coronary intervention (PCI), and coronary artery bypass graft surgery (CABG). The non-invasive procedures studied were exercise treadmill testing, transthoracic echocardiography, and nuclear cardiac imaging [multi-gated acquisition (MUGA), Thallium/Sestamibi]. We examined cumulative incidence of all these procedures during the initial hospital admission for AMI, as well as at 1 year following the date of the initial admission for AMI. We also examined rates of primary PCI, which was defined as receipt of PCI on the day of admission for AMI. In addition, we determined the median waiting period (time in days) between the date of the initial admission and any invasive cardiac procedure received within 1 year.

We also obtained data on prescriptions for medications with anti-ischemic properties, as well as frequency of emergency room and physician visits. The anti-ischemic medications studied were beta-blockers, nitrates and calcium channel blockers. We measured the cumulative incidence of prescriptions filled at 30 days post-discharge. We also measured the cumulative incidence of anti-ischemic combination therapy for the patients who were alive at 1 year post-discharge. Anti-ischemic combination therapy was defined as prescription for one (monotherapy), two (double therapy) or three (triple therapy) agents among beta-blockers, nitrates, and calcium channel blockers at 1 year post-discharge.

The frequencies of emergency room and outpatient physician visits during the year following discharge were also measured. All emergency room visits were included in analyses, irrespective of the reason for consultation. Outpatient physician visits included visits to internists, cardiologists, and family physicians.

### Readmissions and Mortality

Readmission rates for cardiac-specific diagnoses examined were recurrent AMI (ICD-9 code 410), unstable angina (ICD-9 code 411, 413, 414) and congestive heart failure (ICD-9 code 428). Specifically, we measured cumulative incidence of readmission for these diagnoses at 30 days and 1 year following the date of discharge. Consistent with previous studies using similar administrative databases [8], we excluded any elective readmissions for invasive cardiac procedures.

Finally, cumulative incidence of mortality was measured during the initial hospitalization and at 30 days and 1 year after admission.

### Statistical Analysis

We compared demographic and clinical characteristics, use of cardiac procedures and medications, frequency of emergency room and physician visits, and readmission and mortality rates for patients initially admitted for AMI at hospitals with and without on-site cardiac catheterization facilities. We then used multivariable Cox's proportional hazards models to estimate the association between admission at hospitals with and without cardiac catheterization facilities and the study outcomes. Assumptions of proportionality for the Cox models were verified for each study outcome. The models adjusted for age at admission, sex, the interaction between age at admission and sex, the year of admission, co-morbid illnesses (diabetes mellitus, chronic obstructive pulmonary disease, cerebrovascular disease, malignancy, congestive heart failure, renal failure, peripheral vascular disease), shock, specialty of the treating physician (cardiologist or not), university affiliation of hospital of admission, and AMI patient volume of hospital of admission (low, medium or high volume). The study was approved by the institutional review board at the McGill University, Montreal, Canada.

## Results

### Patient Characteristics

Among the 35,289 study patients, 9,355 (27%) were admitted to hospitals with on-site cardiac catheterization facilities. Patients admitted to both types of hospitals had similar demographic and clinical characteristics, but different hospital characteristics (Table 1). For example, patients admitted to hospitals with cardiac catheterization facilities were more likely to be treated by cardiologists, to be admitted to a university-affiliated hospital and a high-volume (>100 AMI admissions/year) hospital. Finally, median length of stay was shorter for patients admitted to hospitals with cardiac catheterization facilities.

### Invasive Cardiac Procedures

Invasive cardiac procedures were performed in a higher proportion of patients admitted to hospitals with cardiac catheterization facilities (Table 2). Catheterization was used more frequently in-hospital [odds ratio (OR), 4.00; 95% confidence interval (CI), 3.78–4.24] and at 1 year (OR, 1.76; 95% CI, 1.68–1.85). Similarly, PCI was used more frequently in-hospital (OR, 6.20; 95% CI, 5.70–6.74) and at 1 year (OR, 1.98; 95% CI, 1.86–2.09). Primary PCI (among patients with catheterization) was also used more frequently (OR, 41.8; 95% CI, 32.2–54.3). Rates of CABG were slightly higher for patients admitted to hospitals with cardiac catheterization facilities in-hospital (OR, 1.91; 95% CI, 1.48–2.45), but modestly lower at 1 year (OR, 0.86; 95% CI, 0.78–0.94).

Waiting periods between admission and procedures were shorter for patients admitted to hospitals with cardiac catheterization facilities. However, for those patients who required CABG following catheterization, there was no difference in the waiting period from catheterization to CABG in both types of hospitals.

### Non-invasive Cardiac Procedures

Overall, non-invasive cardiac procedures were not used more frequently at hospitals without on-site availability of cardiac catheterization facilities (Table 2). To the contrary, echocardiography was used more frequently in hospitals with cardiac catheterization facilities in-hospital (OR, 2.56; 95% CI, 2.43–2.69) and at 1 year (OR, 2.04; 95% CI, 1.93–2.16). Similarly, MUGA imaging was used more frequently in-hospital (OR, 1.32; 95% CI, 1.22–1.42) and at 1 year (OR, 1.24; 95% CI, 1.17–1.32). In contrast, exercise treadmill testing was used similarly in both groups (OR at 1 year, 1.02; 95% CI, 0.91–1.15) and Sestamibi/Thallium imaging was used only slightly less frequently at hospitals with cardiac catheterization facilities (OR at 1 year, 0.93; 95% CI, 0.88–0.98).

### Anti-Ischemic Prescriptions, Emergency Room and Physician Visits

There were no significant differences in rates of prescriptions for cardiac medications at 30 days post-discharge between the two groups (Table 3). Refill rates for sublingual nitrates were also comparable in both groups, as were rates of prescription for anti-ischemic combination therapy at 1 year (Table 3). Furthermore, the total number of emergency room and physician visits was similar in both groups at 1 year post-discharge.

### Readmissions and Mortality

We found similar rates of readmission for recurrent AMI and congestive heart failure at 30 days and 1 year post-discharge in both groups [recurrent AMI and congestive heart failure adjusted hazard ratio (HR), 1.02; 95% CI, 0.89–1.16 and HR, 1.02; 95% CI, 0.90–1.1), respectively] (Table 4). There was no difference in readmission rates for unstable angina (HR, 0.93; 95% CI, 0.85–1.02).

**Figure 1:**
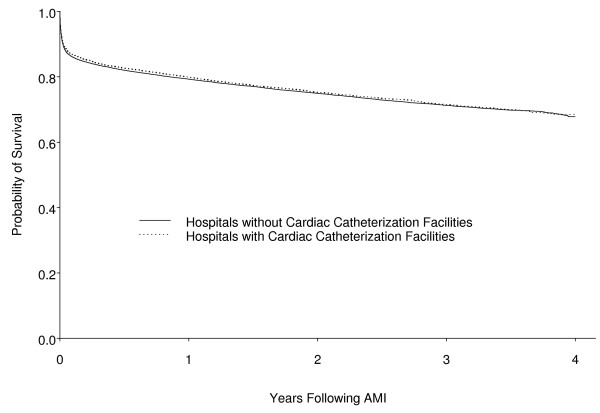
Probability of survival among patients with acute myocardial infarction admitted at sites with and without cardiac catheterization facilities.

Mortality rates were also similar for patients admitted to hospitals with and without cardiac catheterization facilities in-hospital (9% versus 10%), at 30 days (13% versus 14%), and 1 year (20% versus 21%). In addition, adjusted rates were similar for the entire follow-up period (adjusted HR, 0.99; 95% CI, 0.93–1.05) (Figure 1). Finally, because mortality is very high in patients with shock and there was a higher proportion of patients with shock at hospitals with on-site catheterization, we performed a model that excluded patients with shock and failed to find a mortality difference (adjusted HR, 1.0; 95% CI, 0.94–1.07).

In univariate analysis, the effect of physicians specialty, hospital teaching status and volume appeared to also greatly affect the various outcomes that were measures (Table 4). However, the effect of on-site catheterization availability remained the same after addition of these variables to our multivariate models. The relationship between on-site catheterization and mortality and cardiac readmissions is thus unexplained by these other variables. However, in multivariate analysis, cardiologists appears to be associated with better outcomes independent of being admitted to a hospital with on-site catheterization and, in particular, with fewer readmissions for unstable angina [adjusted HR, recurrent AMI: 0.97; 95% CI, 0.86–1.08; congestive heart failure: 0.92; 95% CI, 0.83–1.03; unstable angina: 0.90; 95% CI, 0.83–0.98; mortality: 0.93; 95% CI, 0.88–0.98].

## Discussion

Lack of availability of on-site cardiac catheterization facilities did not result in an increased use of non-invasive testing for risk stratification following AMI. In contrast, despite a higher rate of use of in-hospital catheterization (and presumably left ventriculography), ventricular assessment via echocardiography and MUGA imaging was used more frequently for patients admitted at hospitals with catheterization facilities. Lower use and longer waiting times for invasive cardiac procedures at hospitals without catheterization facilities did not result in worse clinical outcomes. Use of anti-ischemic medications, frequency of emergency room and outpatient physician visits, and rates of readmission for AMI-related cardiac complications and mortality were unaffected by catheterization availability.

The association between availability and use of on-site cardiac catheterization facilities has been well documented [2,3]. Furthermore, the management strategy of routine cardiac catheterization following AMI has been shown not to impact on mortality, but few studies have assessed the impact of this strategy on outcomes such as non-fatal outcomes and utilization of medical resources. A reduction in readmissions for unstable angina was shown in the Thrombolysis In Myocardial Infarction (TIMI) III-B trial, thus slightly favoring the "early invasive" approach [15]. More recently, the Treat Angina with Aggrastat and Determine Cost of Therapy with an Invasive or Conservative Strategy-Thrombolysis In Myocardial Infarction 18 (TACTICS-TIMI 18) investigators reported a lower incidence of major cardiac events with the use of an "early invasive" strategy combined with glycoprotein IIb/IIIa inhibition [22]. Whereas the TIMI-IIIB and TACTICS trials prospectively examined the incidence of cardiac events following unstable angina and AMI, few investigators have used large administrative databases to examine non-fatal outcomes and resource utilization with a population-based approach. Our study extends these previous studies by providing new information, at the population level, for a large number of patients from a recent cohort. This type of analysis is complementary to that of the clinical trials, as it is relevant to the "real world" care of patients in several jurisdictions where the routine application of the invasive approach is not feasible. However, given the overall relatively infrequent use of the invasive approach in both study groups, this study cannot provide a valid comparison of a routine invasive versus non-invasive approach. Instead, it provides evidence that certain outcomes are similar for patients treated at hospitals with more versus less selective use of invasive cardiac procedures, albeit at an overall still highly selective level. The overall equivalence for other important endpoints, such as outpatient burden of angina and heart failure symptoms, remains indeterminate.

Only one other study has recently used administrative data to examine such a hypothesis [8]. Alter et al. found that the non-fatal composite 5-year event rate was lower when patients were admitted to hospitals with cardiac catheterization facilities from 1992 to 1993. Unlike Alter et al., we did not find an effect of availability of cardiac catheterization facilities on rates of readmissions for unstable angina. However, only 11% of patients in the study by Alter et al. were initially admitted to hospitals with cardiac catheterization facilities, suggesting that lower readmission rates for angina may have been related to elective admissions for invasive cardiac procedures. Our results regarding cardiac readmissions reflect a more recent trend of practice.

In order to identify the interplay between physician specialty, university affiliation, volume of AMI admissions and on-site availability of cardiac catheterization facilities, we included all these variables in our multivariate models. There appears to be little confounding bias by physician specialty, hospital teaching status and hospital volume of AMI admissions. We could not identify any apparent benefit of being treated at hospitals with cardiac catheterization facilities, despite the more prevalent use of invasive cardiac procedures, the shorter waiting periods for revascularization, the greater proportion of patients treated by cardiologists, university-affiliations, and the higher volume of AMI admissions. However, our multivariate regression estimates should be interpreted with caution due to their high correlation with availability of on-site cardiac catheterization.

Our study presented the following limitations. First, while we adjusted for several important patient-, physician- and hospital-related characteristics in our multivariate analyses, our administrative databases do not contain information on in-hospital pharmacological treatment including thrombolytic therapy or detailed clinical characteristics that could confound our analyses. However, the similarities in available clinical characteristics across the two groups provide a good indication that more detailed clinical characteristics would also be similar. Findings from a previous study using detailed chart review data support this claim. Second, it was difficult to assess the role of the treating physician and the teaching status and volume of the admitting hospital in relationship with catheterization laboratory availability because these variables are highly correlated. The difficulty to abstract collinear variables in such analyses represents an important challenge in this field. Finally, there is the possibility for some misclassification bias in our databases. For example, a readmission diagnosis of unstable angina may actually reflect an elective admission for pre-planned cardiac catheterization. As in previous studies, we excluded all readmissions where catheterization was performed on the same or next day following admission. In fact, few patients were excluded under this criterion; we repeated analyses with and without these exclusions and found comparable readmission rates.

## Conclusion

In conclusion, the lower use of invasive cardiac procedures at hospitals without on-site catheterization did not translate into a higher use of non-invasive tests. Furthermore, use of anti-ischemic cardiac medications, emergency room visits, outpatient physician visits, readmissions for AMI-related complications and mortality were similar at each type of hospital.

## Competing interests

The author(s) declare that they have no competing interests.

## Authors' contributions

AH drafted the manuscript. CB assisted with the literature review and editing of the manuscript, as well as the statistical analyses. HR performed the statistical analyses. ME and LP conceived of the study and obtained the study data. All authors participated in the design and coordination of the study, and read and approved the final manuscript.

## Pre-publication history

The pre-publication history for this paper can be accessed here:



## Figures and Tables

**Table 1:** Patient and hospital characteristics, according to availability of on-site cardiac catheterization facilities at the hospital of admission for acute myocardial infarction

	**Hospitals with Catheterization Facilities (n= 9,355)**	**Hospitals without Catheterization Facilities (n= 25,934)**
Patient characteristic (%)		
Median age, years (IQR)	67 (55–76)	67 (55–76)
Patients >65 years	56.2	56.3
Male	65.4	64.9
Hypertension	35.0	28.8
Diabetes mellitus	22.0	19.9
Dyslipidemia	20.9	17.8
Heart failure	21.7	22.1
Atrial fibrillation	9.9	8.7
Chronic obstructive pulmonary disease	12.2	15.2
Peripheral vascular disease	8.5	7.8
Cerebrovascular disease	6.8	6.4
Chronic renal failure	7.3	5.7
Acute renal failure	4.8	4.6
Shock	5.7	4.7
Malignancy	2.1	2.3
Hospital characteristic (%)		
Median length of stay, days (IQR)	7 (5–12)	9 (6–14)
Treating physician specialty		
Cardiology	92.4	28.9
Family medicine	2.8	57.7
Internal medicine	2.3	12.4
University-affiliation	86.9	33.6
AMI patient volume		
< 50 cases AMI/year	0	1.2
50–100 cases AMI/year	0	17.2
> 100 cases AMI/year	100.0	81.6

IQR denotes interquartile range, AMI denotes acute myocardial infarction.

**Table 2:** Cumulative incidence of invasive and non-invasive cardiac procedures

	**Hospitals with Catheterization Facilities (n = 9,355)**	**Hospitals without Catheterization Facilities (n = 25,934)**
Non-invasive procedures (%)		
Exercise treadmill test *		
In-hospital	27.2	24.0
1 year	48.2	47.5
Echocardiogram		
In-hospital	66.7	44.0
1 year	76.2	61.0
MUGA scan		
In-hospital	11.0	8.6
1 year	23.9	20.1
Thallium/Sestamibi scan		
In-hospital	8.3	6.0
1 year	23.8	25.2
Invasive procedures (%)		
Catheterization		
In-hospital	35.1	11.9
1 year	51.2	37.3
PCI		
Primary PCI	9.0	0.2
In-hospital	18.5	3.5
1 year	28.2	16.6
CABG		
In-hospital	1.1	0.6
1 year	8.2	9.4

MUGA denotes multi-gated acquisition, PCI denotes percutaneous coronary intervention, CABG denotes coronary artery bypass graft surgery.

*Data available only for patients admitted from June 1, 1998–1999 (n = 8,741).

**Table 3:** Cardiac prescriptions, physicians and emergency room visits post-discharge after acute myocardial infarction

	**Hospitals with Catheterization Facilities (n = 4,116)**	**Hospitals without Catheterization Facilities (n = 11,190)**
Medication class* (%)		
Aspirin	65.4	64.2
Beta-blockers	57.7	52.6
ACE inhibitors	45.2	44.0
Nitrates (any form)	72.7	73.0
Calcium channel blockers	27.6	28.1
Lipid-lowering agents	24.9	20.8
Anti-ischemic combination therapy at 1 year^† ^(%)		
Monotherapy	38.8	41.6
Double therapy	27.3	27.9
Triple therapy	7.8	7.2
Refills for sublingual nitrates at 1 year (%)	31.4	28.7
Emergency room visits at 1 year (%)		
0 visits	43.3	43.3
1–3 visits	41.4	41.3
≤4 visits	15.4	15.4
Physician visits at 1 year (%)		
0–3 visits	22.3	18.9
4–8 visits	38.5	39.7
≤9 visits	39.2	41.4

ACE denotes angiotensin-converting enzyme.

* For patients ≤65 years of age only; ^† ^Anti-ischemic combination therapy was defined as prescription for one (monotherapy), two (double therapy) or three (triple therapy) agents among beta-blockers, nitrates, and calcium channel blockers at six months post-discharge (for patients ≤65 years of age only, n = 15,306).

**Table 4:** Effect of catheterization availability, physician specialty, university affiliation and volume on fatal and non-fatal outcome

	**Hazard Ratios and 95% Confidence Intervals**			
	
	**Catheterization Availability**	Physician Specialty*	**University Affiliation**	**Volume >= 150**
**Congestive Heart Failure**				
Unadjusted	1.01 (0.92 – 1.12)	0.85 (0.78 – 0.92)	1.03 (0.94 – 1.12)	0.97 (0.88 – 1.06)
Adjusted	1.02 (0.90 – 1.15)	0.92 (0.83 – 1.03)	1.07 (0.96 – 1.19)	0.94 (0.85 – 1.05)
**Unstable Angina**				
Unadjusted	0.81 (0.76 – 0.87)	0.82 (0.77 – 0.87)	0.91 (0.86 – 0.97)	0.758 (0.71 – 0.81)
Adjusted	0.93 (0.85 – 1.02)	0.90 (0.83 – 0.98)	1.11 (1.03 – 1.19)	0.80 (0.74 – 0.86)
**Recurrent AMI**				
Unadjusted	0.97 (0.87 – 1.07)	0.90 (0.83 – 0.99)	0.92 (0.84 – 1.01)	0.93 (0.84 – 1.03)
Adjusted	1.02 (0.89 – 1.16)	0.97 (0.86 – 1.08)	0.92 (0.83 – 1.03)	0.95 (0.85 – 1.06)
**Mortality**				
Unadjusted	0.98 (0.94 – 1.03)	0.84 (0.81 – 0.88)	1.02 (0.98 – 1.06)	1.05 (1.00 – 1.10)
Adjusted	0.99 (0.93 – 1.05)	0.93 (0.88 – 0.98)	1.02 (0.97 – 1.07)	1.08 (1.03 – 1.14)

* Cardiologists compared to internists or family physicians.

Note: The adjusted relative risks need to be interpreted with caution because variables are highly correlated.
